# Modeling system states in liver cells: Survival, apoptosis and their modifications in response to viral infection

**DOI:** 10.1186/1752-0509-3-97

**Published:** 2009-09-22

**Authors:** Nicole Philippi, Dorothee Walter, Rebekka Schlatter, Karine Ferreira, Michael Ederer, Oliver Sawodny, Jens Timmer, Christoph Borner, Thomas Dandekar

**Affiliations:** 1 Dept of bioinformatics, Biocenter, Am Hubland, University of Würzburg, 97074 Würzburg, Germany; 2 Institute of Molecular Medicine and Cell Research (ZBMZ), Albert Ludwigs University Freiburg, 79104 Freiburg, Germany; 3 Institute for System Dynamics, University of Stuttgart, Pfaffenwaldring 9, 70569 Stuttgart, Germany; 4 Freiburger Zentrum für Datenanalyse und Modellbildung (FDM), Albert Ludwigs University Freiburg, 79070 Freiburg, Germany

## Abstract

**Background:**

The decision pro- or contra apoptosis is complex, involves a number of different inputs, and is central for the homeostasis of an individual cell as well as for the maintenance and regeneration of the complete organism.

**Results:**

This study centers on Fas ligand (FasL)-mediated apoptosis, and a complex and internally strongly linked network is assembled around the central FasL-mediated apoptosis cascade. Different bioinformatical techniques are employed and different crosstalk possibilities including the integrin pathway are considered. This network is translated into a Boolean network (74 nodes, 108 edges). System stability is dynamically sampled and investigated using the software SQUAD. Testing a number of alternative crosstalk possibilities and networks we find that there are four stable system states, two states comprising cell survival and two states describing apoptosis by the intrinsic and the extrinsic pathways, respectively. The model is validated by comparing it to experimental data from kinetics of cytochrome c release and caspase activation in wildtype and Bid knockout cells grown on different substrates. Pathophysiological modifications such as input from cytomegalovirus proteins M36 and M45 again produces output behavior that well agrees with experimental data.

**Conclusion:**

A network model for apoptosis and crosstalk in hepatocytes shows four different system states and reproduces a number of different conditions around apoptosis including effects of different growth substrates and viral infections. It produces semi-quantitative predictions on the activity of individual nodes, agreeing with experimental data. The model (SBML format) and all data are available for further predictions and development.

## Background

The decision to undergo apoptosis is complex, ultimately fatal for the cell and yet important for continued health and proper regeneration of most tissues. As an example of high medical relevance we investigate here the liver tissue. A number of different studies already investigated and modeled different pathways implicated in liver cell apoptosis [1-4]. We have started our analysis from an interesting observation in cultured hepatocytes: Depending on the culture conditions, different pathways for apoptosis are used. In vivo, hepatocytes use the so-called type II signaling pathway to apoptosis after stimulation with Fas ligand (FasL). However, in the presence of a collagen I monolayer, primary mouse hepatocytes switch to the type I signaling pathway in response to FasL. Whereas type II uses mitochondrial signaling implying tBid formation and cytochrome c release for effector caspase-3 activation, the type I pathway is more direct, bypassing the mitochondrial components. When the cells are kept in suspension right after isolation, the signaling does not switch but stays type II as in vivo [5]. We therefore inferred that this switch most likely occurs due to a crosstalk between the apoptosis and the collagen-integrin signaling pathways. This prompted us to conduct a systematic search for different system states in liver cells regarding apoptosis.

This study aims at integrating different apoptosis pathways (including those investigated in the studies cited above) and considering a number of different crosstalk possibilities in hepatocytes including different members of the integrin pathway. The complete established network is first investigated by sequence and domain analysis [see Additional File 1] and then turned into a Boolean network of activating and suppressing nodes. As for many nodes of this network exact parameters or measurements are not available, a global system stability analysis has been performed applying the software SQUAD [6]. We show that the complete network comprises four stable system states; two states of cell survival and two states of apoptosis. In a next step, the model is qualitatively validated by experimental readout data such measuring caspase activities by fluorogenic substrates. It includes and reproduces the system response behavior after modifications, such as knocking out the BH3-only protein Bid or infecting the cells with cytomegalovirus.

Overall, the presented model provides a comprehensive system overview of the complex apoptosis signaling network, gives insights into its structural properties and can serve as a first basis for more detailed kinetic modeling of the complete network of signaling molecules in hepatocytes.

## Results

### Modeling

**Figure 1:**
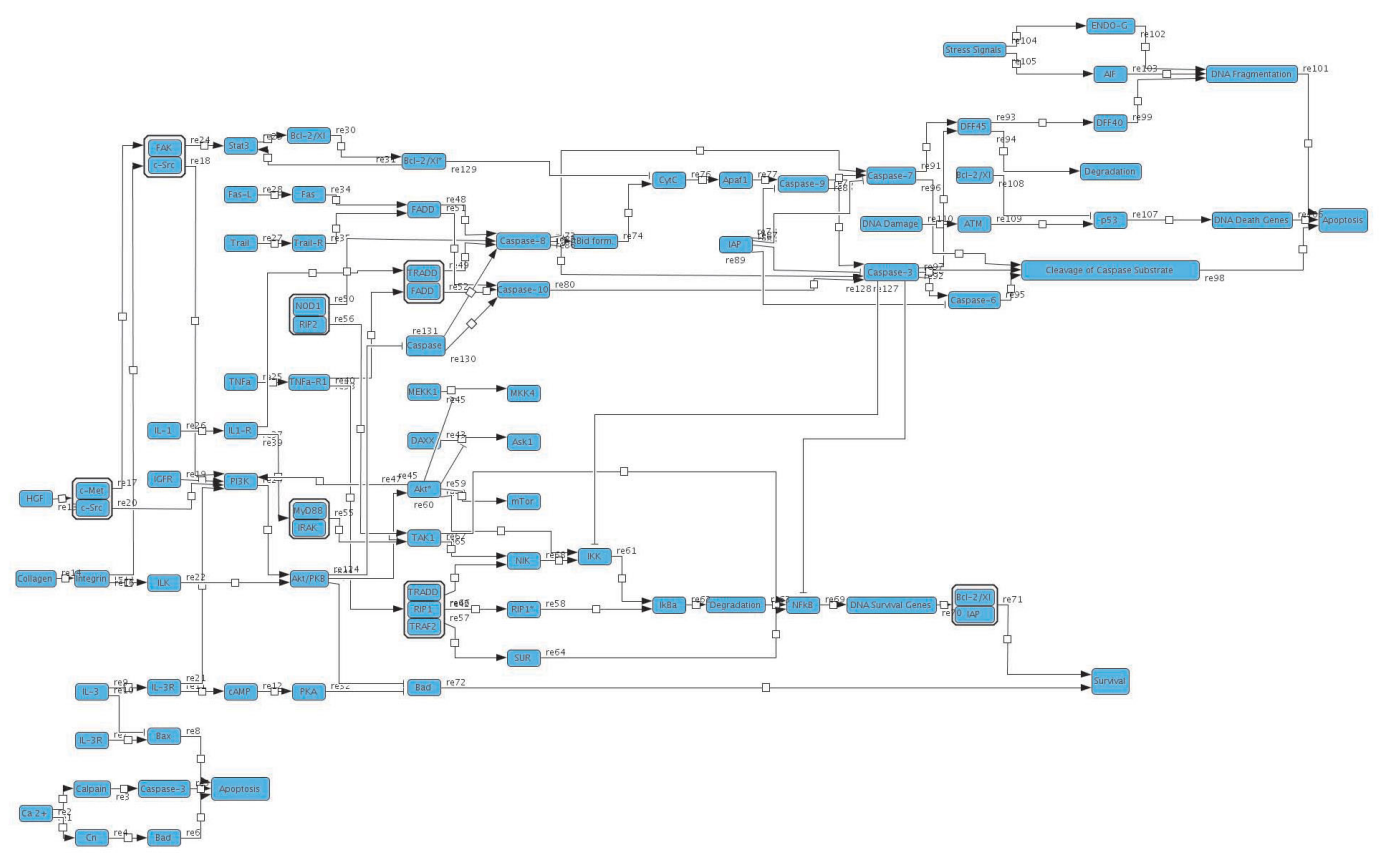
**Schematic view of major apoptosis pathways in mammalian cells**.Comprehensive network of proteins (activating, inhibiting) involved in liver cell apoptosis including different nodes around key pathways of apoptosis in hepatocytes as well as a number of proteins implicated in crosstalk.

For a systematic investigation network data from genome information, databases, sequence- and domain analysis as well as network module analysis were compiled. We established a comprehensive network of proteins involved in the apoptosis signaling of hepatocytes including different nodes around key pathways of apoptotic signaling as well as a number of proteins implicated in crosstalks (overview in Figure 1; details and interaction maps for all the steady states in suppl. Material). Additional file 2: Table 1S lists all involved proteins, their sequences as well as their domain compositions, labeling key domains for apoptosis, for kinase signaling and executioner caspases. These interactions were taken into account in the resulting model.

The network allows a systematic simulation and investigation of different system states. This also implies all crosstalk possibilities because the hepatocyte system may switch from life to apoptosis via different pathways and system states. Table 1 lists the different tested versions of the network. For each topology the program SQUAD was used to systematically test the number of stable states in the network.

**Table 1:** Overall view of tested hepatocyte apoptosis models

**Model**	**Description**	**Nodes**	**Edges**	**Steady States**	**Feedback Loops**	**State**
**Model A (All)**	All interactions and crosstalk possibilities considered. These include: additional component as a pos. feedback between Akt/PKB and PI3K, additional component as a pos. feedback between Bcl-2/x_L _and Stat3, additional Akt substrates	74	108	4	2 positive feedback loops	Apoptosis Survival Apoptosis Survival

Model 1	reduced core model (only components listed in official databases)	66	94	1	no feedback loop	Apoptosis

Model 2	Core model + ILK pathway and Akt/PKB*	68	100	2	1 positive feedback loop	Apoptosis Survival

Model 3	Core model + TRADD/FADD interactions and ILK pathway	68	100	1	no feedback loop	Apoptosis

Model 4	Core model + Akt/PKB* and PI3K pathway	68	100	2	1 positive feedback loop	Apoptosis Survival

**Model C (Core)**	**Core model**	67	99	1	no feedback loop	Apoptosis

Model 5	Model All with a Bid KO	74	107	4	2 positive feedback loops	Apoptosis Survival Apoptosis Survival

Model 6	Model All + cytomegalovirus infection (influence of M45)	75	110	4	2 positive feedback loops	Apoptosis Survival Apoptosis Survival

Model 7	Model All + cytomegalovirus infection (influence of M36)	75	110	4	2 positive feedback loops	Apoptosis Survival Apoptosis Survival

Model 8	Model All + cytomegalovirus infection (influence of M36 and M45)	76	111	4	2 positive feedback loops	Apoptosis Survival Apoptosis Survival

^1^Further models tested in detail; additional feedback/forward loops to the core model C: These are all included in model A. Modifications for Model C to get Model A: additional nodes: Akt*, Collagen, Integrin, M45, DAXX, ask1, MKK4, MEKK1, mTor, RIP*, Bcl-2/xL*; removal of the following nodes: Bak/Bax, CytC

The program SQUAD starts from a Boolean network model of the system and subsequently uses a heuristic algorithm for parameter estimation and systematic parameter variation to count and collect all stable system states. SQUAD turns the Boolean network into a dynamic simulation, replacing the Boolean states for each node of either 0 (off) or 1 (on) by a hysteresis curve to interpolate all intermediate states for each node starting there with similar basic assumptions for the exponential function [see Additional file 2 for complete equation]. Briefly the exponent h is set to 0,5 as default, but then consecutively modified according to network connectivity for each specific node. The space is already in the Boolean network equal to 2^(number of nodes) ^for different network states, i.e. for the full network with 74 nodes this corresponds to 2^74 ^states or roughly 10^23 ^states. This algorithm (SQUAD) efficiently implements a reduced order binary decision diagram [7] and systematically explores the large solution space. Table 1 shows that depending on the number and kind of feedback loops there are networks with one, two or four different system states. A positive feedback loop doubles the amount of steady states and a negative feedback loop is responsible for the stabilization of a steady state.

Twenty different versions of the model were analyzed [see Additional file 2]. The complete model (A, all) shows four different steady states (Table 2), characterized by additional components such as positive feedback loops between Akt/PKB and PI3K, Bcl-2/x_L _and Stat3, as well as by additional Akt substrates. To test for robustness, a Bid KO model containing 73 nodes was tested and the same 4 steady states were obtained, yet modified in some of the final concentrations (Table 3; compare with experimental data on collagen shown later). Finally, based on these results three models representing different viral infections were additionally constructed (Table 1).

**Table 2:** Steady state analysis for hepatocyte apoptosis Model A (complete model)^1^

**Model A** **Steady State A1**	**Value**	**Model A** **Steady State A2**	**Value**	**Model A** **Steady State A3**	**Value**	**Model A** **Steady State A4**	**Value**
Akt*	**0**	Akt*	**0**	Akt*	**0,99**	Akt*	**0,99**
Akt/PKB	**0**	Akt/PKB	**0**	Akt/PKB	**0,79**	Akt/PKB	**0,79**
Apaf1	**1**	Apaf1	**0**	Apaf1	**0**	Apaf1	**0**
Apoptosis	**0,98**	Apoptosis	**0,98**	Apoptosis	**0**	Apoptosis	**0**
Bcl-2/Xl	**0**	Bcl-2/Xl	**1**	Bcl-2/Xl	**0**	Bcl-2/Xl	**1**
Bcl-2/Xl*	**0**	Bcl-2/Xl*	**1**	Bcl-2/Xl*	**0**	Bcl-2/Xl*	**1**
Caspase-10	**0,85**	Caspase-10	**0,85**	Caspase-10	**0**	Caspase-10	**0**
Caspase-3	**0,99**	Caspase-3	**0,95**	Caspase-3	**0**	Caspase-3	**0**
Caspase-6	**1**	Caspase-6	**1**	Caspase-6	**0**	Caspase-6	**0**
Caspase-7	**1**	Caspase-7	**0,83**	Caspase-7	**0**	Caspase-7	**0**
Caspase-8	**0,78**	Caspase-8	**0,78**	Caspase-8	**0**	Caspase-8	**0**
Caspase-9	**1**	Caspase-9	**0**	Caspase-9	**0**	Caspase-9	**0**
Cleavage	**1**	Cleavage	**1**	Cleavage	**0**	Cleavage	**0**
CytC	**1**	CytC	**0**	CytC	**0**	CytC	**0**
DFF40	**1**	DFF40	**1**	DFF40	**0**	DFF40	**0**
DFF45	**1**	DFF45	**1**	DFF45	**0**	DFF45	**0**
Fragmentation	**0,85**	Fragmentation	**0,85**	Fragmentation	**0**	Fragmentation	**0**
Survival Genes	**0**	Survival Genes	**0**	Survival Genes	**0,99**	Survival Genes	**0,99**
Degradation	**0,93**	Degradation	**0,93**	Degradation	**0,9**	Degradation	**0,9**
Caspase	**1**	Caspase	**1**	Caspase	**0,01**	Caspase	**0,01**
IKK	**0**	IKK	**0**	IKK	**0,93**	IKK	**0,93**
IkBa	**0**	IkBa	**0**	IkBa	**0,91**	IkBa	**0,91**
NFkB	**0**	NFkB	**0**	NFkB	**0,79**	NFkB	**0,79**
PI3K	**0**	PI3K	**0**	PI3K	**0,73**	PI3K	**0,73**
Stat3	**0**	Stat3	**0,93**	Stat3	**0**	Stat3	**0,93**
Survival	**0**	Survival	**0**	Survival	**0,93**	Survival	**0,93**
mTor	**0**	mTor	**0**	mTor	**1**	mTor	**1**
s69	**0**	s69	**0**	s69	**1**	s69	**1**
tBid	**0,98**	tBid	**0,98**	tBid	**0**	tBid	**0**

^1^All key nodes of the network are listed together with the activation state for each steady state.

Model A was chosen for further investigations, as it nicely captures the richness of different apoptotic states and agrees with experimental data (see below). There are four different system states. Steady state A1 represents the mitochondrial/intrinsic pathway like it is for cells in suspension. Bcl-2 is inactive while all caspases are active, additionally showing a cytochrome c release into the cytoplasm. Steady state A2 shows the extrinsic pathway which correlates to cells grown on collagen. Bcl-2 is active but it's not interfering in the extrinsic pathway. State A3 and state A4 mimic stable living cells. In both states AKT and NFκB are active. The difference between these two states is the activation or inactivation of Bcl-2 and Stat3. The choice between both system modes should be cell type dependent, for instance regulation by the JAK/STAT3 pathway and resulting Bcl-2 expression control in the non-apoptotic state is known for hematopoetic cells [8].

### Verification and biological data

**Figure 2:**
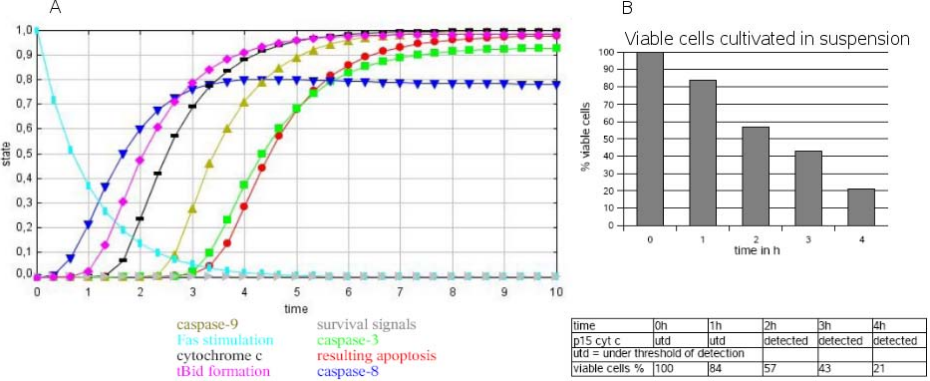
**Apoptosis after FasL stimulation in hepatocytes cultured in suspension (shown on the left panels)**. A. SQUAD simulation of apoptosis in hepatocytes. Activation values for network nodes are calculated and translated into concentration predictions. Maximum value is 1, minimum value is 0. Color code: Concentration curves for survival signals (grey), caspase-9 (beige), caspase-8 (dark blue), caspase-3 (green), cytochrome c release into the cytosol (black), resulting apoptosis (red), tBid formation (pink), Fas stimulation (light blue). B. Experimental data. These are given in the inserted table. Primary hepatocytes are kept in suspension and treated with FasL direct after isolation. The values for the viable cells are shown in addition in the histogram. Wildtype hepatocytes are cultured in suspension and treated with 50 ng/ml N2A FasL for 0-4 hours. Cytosolic lysates were analyzed by Western Blotting and probed against cytochrome c (p15). Note that cytochrome c is strongly released into the cytosol after 2 hours FasL treatment in suspension hepatocytes.

**Figure 3:**
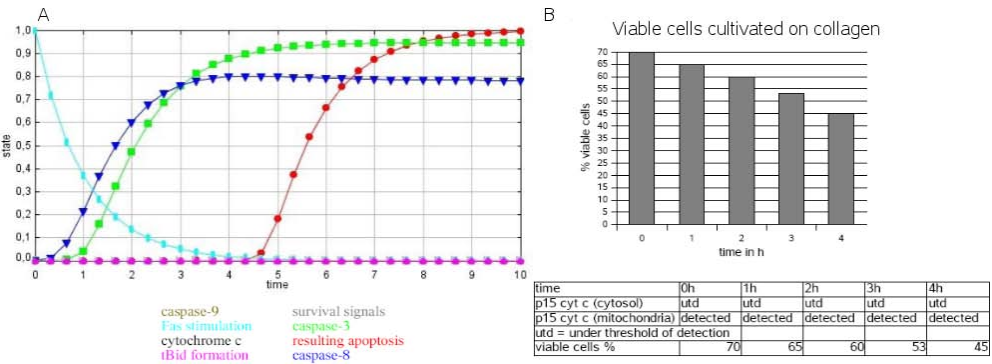
**Apoptosis after FasL stimulation in hepatocytes grown on collagen (shown on the right panels)**. A. SQUAD simulation of apoptosis in hepatocytes. Activation values for network nodes are calculated and translated into concentration predictions. Maximum value is 1, minimum value is 0. Color code: Concentration curves for survival signals (grey), caspase-9 (beige), caspase-8 (dark blue), caspase-3 (green), cytochrome c release into the cytosol (black), resulting apoptosis (red), tBid formation (pink), Fas stimulation (light blue). B. Experimental data. Wildtype hepatocytes are cultured on collagen I and treated for 0-6 hours with 50 ng/ml N2A FasL. Lysates were fractionated into cytosol and mitochondria and were subjected to cytochrome c Western Blot analysis. Note that in collagen cultured hepatocytes, all cytochrome c remains in the mitochondria and is not released into the cytosol upon FasL treatment. Experimental data is given in the table. FACS analysis of GFP-annexin-V/PI stained wildtype primary hepatocytes plated on collagen, treated with 50 ng/ml N2A FasL for 0 to 4 hours. The values for the viable cells are given in a histogram.

The signaling behavior of hepatocytes in suspension (Figure 2) or grown on collagen (Figure 3) correlates nicely with the system switches predicted from the SQUAD simulation. SQUAD allows the systematic collection and comparison of stability states of the system as well as transitions between them. The input for a respective node, i.e. all interactions leading to its activation (1) or inactivation (0), is systematically sampled and a decay term for its inactivation is included (see also Materials and Methods). When hepatocytes are stimulated with FasL (the node changed from an inactive to an active ligand state), the system simulates apoptosis starting from FasL stimulation. In the model for growth on collagen, cytochrome c is not released (no activation by tBid, inhibition by Bcl-2/Xl) and apoptosis is delayed for both apoptosis nodes in the model.

In each upper panel (Figure 2A, Figure 3A) the behavior of the key system nodes of the network is depicted in relation to time, representing the downstream molecular components of the signaling pathway, i.e. stimulation with FasL, survival signals, caspase-3, caspase-8, caspase-9, tBid formation, cytochrome c release and finally apoptosis induction. The values for system node states range between 0 (inactive) and 1 (active). The equations for the dynamic simulations yield time courses which reproduce the experimental data well. Time units are set to hours.

The comparison of the calculated curves (Figure 2A) for the expected readout with the observed readout shows a good qualitative agreement (Figure 2B). Both also agree quantitatively if the time scale is set to hours in the upper panel. Maximal stimulation is reached after 4 hours both by simulation and experiment (Figure 2). Whereas Figure 2A displays the behavior of the network for FasL stimulated primary hepatocytes cultured in suspension, Figure 3A depicts that for the cells grown on collagen. We show for the latter condition that the dynamics modeled by our SQUAD simulation involving the A2 system state (apoptosis on collagen, crosstalk inhibits mitochondrial pathway for apoptosis, Figure 3A, top) is well compatible with the experimental readout from experiments with fluorogenic caspase substrates (Figure 3B, bottom) if the time scale is again set to hours in the upper panel (Figure 3A, simulation). Note the difference in the tBid curves, because while there is an increase of tBid formation in Figure 2A, the tBid values value were at baseline. This is in accordance with experimental data on the system [5].

Apoptosis was defined by the decline of the number of surviving cells over time (Figure 2A, Figure 3A). Some of the cells cultivated on collagen are predicted to be viable by this model. This corresponds to the experimentally determined system read-out (Figure 2B and Figure 3B). The delay of apoptosis experimentally observed for cells cultivated on collagen could also be modeled by a delay for apoptosis in the simulation. The exact values for the experimental data are given by Walter et al. [5]: Cytochrome c release is represented by a black line and was measured by Western blotting. We carried these experiments out ourselves (DW, KF, CB) [see Additional file 2: Experimental procedures]. In suspension there is prolonged release of cytochrome c and apoptosis. In contrast, growth on collagen does not lead to release of cytochrome c into the cytoplasm [see Additional file 2: Experimental procedures]. The model reproduces this correctly. For suspension cells cytochrome c is released (Figure 2A), for growth on collagen cytochrome c is at baseline and no release of cytochrome c is obtained (Figure 3A). Both in the experiment and the model (Figure 2) cytochrome c is already strongly released into the cytosol of suspension grown hepatocytes after 2 hours of FasL treatment, reaching a maximum after 4 hours (typical type II pathway as *in vivo*). Collagen cultured hepatocytes, dying after 4 hours according to experimental data, retain cytochrome c in the mitochondria and do not release it into the cytosol upon FasL treatment (Figure 3B), as is typical for the type I pathway. Caspase-8 activity reaches a maximum in suspension cells after 3 hours of FasL treatment and stays high throughout the experiment (Figure 2A). In collagen cultured cells (Figure 3A) caspase-8 activity reaches a plateau after increasing for 4 hours. Our model is qualitative as it does not include direct kinetic parameter input but instead an exponential curve estimate by SQUAD to interpolate between inactive and active state for each network node. However, the calculated values allow ratio estimates between the activities of different nodes and, obviously, whether they inhibit or promote signals in the network. In particular, the qualitative behavior over time (Figure 3A) including different behavior for different nodes is overall correctly simulated and agrees with the experimental observations such as the number of surviving cells or cytochrome c release (Figure 3B). The model also fits to further experimental data available on the qualitative differences concerning tBid formation, caspase-8 activation, the release of some cytochrome c after about 3 hours [5] and caspase-9 activity levels which first increase below the value in Figure 3A, but then return to zero.

SQUAD samples the whole system and its stability which is the key for biological system insights and thereby outweighs that it is semi-quantitative. Individual subnetworks do not offer the integrated system view and analysis. Including these data this can be refined using quantitative models with specific ODEs for each component. However, this requires detailed parameter estimates often not (yet) available. Moreover, SQUAD additionally allows for a systematic exploration of other influences which lead to different system states.

### Simulation of a viral infection

As an example for medical application we investigated the blocking of cell death by viral interference. We used model A and added key nodes interacting under cellular infection by cytomegalovirus. The interacting viral proteins are M36 and M45 [9-11]. Such viral protein interventions (a pathological way of crosstalk) are a good example for interference with the system pathways by many conceivable ways including also therapeutic intervention such as siRNAs and different drugs. Again, the steady state analysis following these modifications shows a system with four steady states if neither M36 nor M45 are activated or expressed.

**Figure 4:**
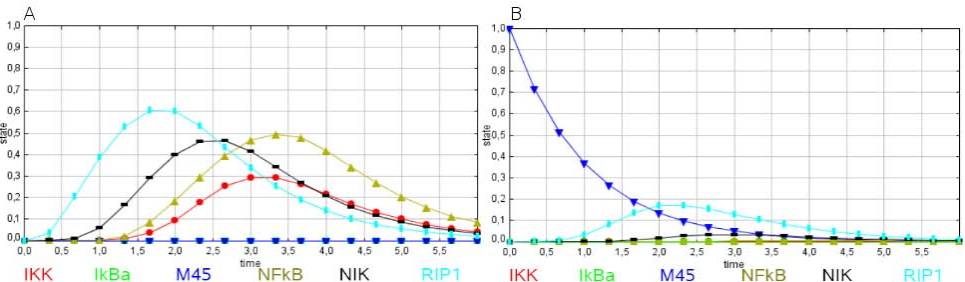
**Viral infection in hepatocytes**. SQUAD simulation data of hepatocytes after TNFα stimulation. M45 interacts with RIP1 and suppresses TNFα-induced NFκB activation. A. SQUAD simulation data of non infected hepatocytes. TNFα (15 min) induced rapid (30 min [17]) NFκB activation. Detailed data from experiment are given in [10]. B. SQUAD simulation data of hepatocytes under influence of viral infection. TNFα mediated IκBα degradation (5 min [18]) was blocked in cells expressing M45. Detailed data from experiment are given in [11]. Activation values for network nodes are calculated and translated into concentration predictions. Maximum value is 1, minimum value is 0. Color code: Concentration curves for RIP1 (cyan), M45 (blue), IκBα (green), NIK (black), NFκB (beige), IKK (red).

However, when M36 or M45 are activated, only the blocked apoptotic, continuous viable state remains. There are two different ways to establish this system state:

(i) The cytomegalovirus protein M36 blocks the mitochondrial apoptosis pathway by enhancing the survival function of Bcl-2. The stable system state corresponding to this inhibition is shown in Additional file 2: Table 4S and predicts the following nodes to be active/inactive: Bcl-2/x_L _(active), Bid, Casp-10, Casp-3, Casp-6, Casp-7, Casp-8, Casp-9, DFF40, DFF45 (all inactive). Also this system state nicely correlates with data from the literature [9-11].

(ii) M45 (blue in Figure 4) blocks the apoptotic pathway via RIP1 (cyan, Figure 4), which is a crucial protein kinase of the death receptor complex. M45 blocks the mono-ubiquitination of RIP1, which results in a second viral intervention to prevent apoptosis in the infected cell if the first break (enhancing the survival function of Bcl-2) is circumvented by the host defense. This activation of an alternative apoptosis block is simulated by our model (Figure 4A hepatocytes without virus versus Figure 4B with the virus). M45 blocks in Figure 4B the TNFα-induced, caspase-independent cell death by binding to RIP1 and binding does not require TNF-R1 or TRAF2/5 as shown previously [11]. M45 inhibits caspase-independent cell death and as a consequence also the activation of p38 MAPK. Furthermore, the predicted readout (e.g. NFκB, IκBα) fits well to experimental data obtained during viral infection (e.g. Figure 3, pp 3095-3096, [11]). Note again that no parameter fitting to the experimental values was done or used for the time course prediction.

## Discussion

### System behavior

The hepatocyte provides a fascinating and complex model to investigate cellular system states concerning apoptosis and survival or proliferation. In several previous studies on this topic, the authors either looked at the dynamics of signaling cascades [1,4], at individual key signaling switches [12] or at the possibility of a bistable apoptosis system regarding caspase activation and the roles of Bax, Bcl-2 and mitochondrial transition pores [2,3]. Here we have chosen a different approach by first investigating system topology and individual nodes to establish a Boolean network of the interacting components and then turning these interactions into a dynamical simulation to answer which stable system states are possible.

We started our analysis with FasL-mediated apoptosis signaling in primary hepatocytes. The network was then extended and systematically explored. In particular, signaling crosstalks triggered by different growth conditions, cell-cell contacts and the attachment to collagen monolayers were included. The survival signaling kinase AKT and its up- and downstream partners PI3K, mTor, MEKK1 and DAXX were considered. The inhibitory role of AKT on apoptosis induction agrees well with recent cell biology data [13]. Furthermore we looked at sub-networks acting during early phases of FasL-mediated signaling (Fas, FasL, FADD, caspase-8), during the effector phase of apoptosis (XIAP, caspase-3 and feedback towards caspase-8), at branching points between apoptosis and proliferation (NFκB, IκBα, SUR, IKK), within the caspase signaling cascade (caspase-10, caspase-3, caspase-6), during type II death receptor signaling (caspase-8, tBid formation, caspase-9, Bcl-2/x_L_) and during viral interference of apoptosis (proteins M36 and M45 from cytomegalovirus). Thereby, we established an overview of surrounding network interactions modifying the classical FasL-mediated apoptosis pathway. Besides network topology this step of the analysis also included all protein sequences involved as well as their specific domain compositions.

By applying the simulation tool SQUAD this network of nodes was next turned into a Boolean network. As detailed kinetic data on these numerous interacting components are not yet available, we instead applied a systems biology approach to study system stability. In particular we systematically studied how many stable system states are available to the system and how these states change with the alterations of the accessible nodes.

As stated above, the full network with 74 nodes could theoretically encode up to 2^74 ^states or roughly 10^23 ^states. However, we could show that in the hepatocyte model there are only four stable system states predicted with reasonable robustness, 2 apoptotic and 2 non apoptotic ones. We tried out different models [see Additional file 2: Table S5] distinguishing between hub nodes (for example tBid and PI3K) and less important nodes (for example NIK). Addition or removal of hub nodes had influence on the system robustness (it did change the number of steady states) as well as an additional interaction between survival and apoptosis (inhibition of each other). Changes of less important nodes had no influence on the systems behavior and these predictions are robust (there is no variation if several nodes are added or removed). Errors in Boolean logic did affect the network output, but again system stability and number and type of stable states did only change if hub nodes were wrongly connected. In steady state A1 we observed activation of the intrinsic mitochondrial pathway of apoptosis, characterized by a caspase-dependent signaling cascade along with caspase-8 mediated tBid cleavage and cytochrome c release. Steady state A2 is also apoptotic but bypasses mitochondrial components and therefore leads to the direct activation of caspase-3 by caspase-8. In state A2, both Bcl-2/x_L _and Stat3 seem to be active, while they are inactive in steady state A1. One of the system's non-apoptotic states is steady state A3. Hereby Akt is active and triggers a survival pathway via NFκB activation and other survival signals that does not lead to apoptosis. In steady state A4, Akt is also active inducing the same survival signaling pathway as in A3, but whereas in A3 Bcl-2/x_L _and Stat3 are inactive, they are active in A4.

### Validation

System transitions which are modeled qualitatively and semi-quantitatively agree well with the direct experimental system read-out data. We can achieve here only semi-quantitative results as no exact kinetics are incorporated in the SQUAD simulation. However, the simulations (Figure 2A, Figure 3A) qualitatively fit the experimental data. Setting the time scale to hours the data even agree semi-quantitatively, e.g. for the time course of cell survival (Figure 2B, Figure 3B) and, as known from further experiments, for the activity of caspase-8 and Bid as well as the growth condition dependent time course of caspase-9 activation. Alternative models applying detailed parameter kinetics and focusing on different sub-networks provide results which nicely correspond to the experimental data. However, these (e.g. [1,3,4]) did not consider different crosstalk possibilities and input from other system nodes (e.g. viral proteins) as detailed and incorporated here. The detailed kinetic SQUAD simulation, its data and the Boolean network are available for any user for further research and detailed comparisons.

After we saw that the model at least qualitatively encapsulates central system states, is robust, and agrees semi-quantitatively with experimental data, we next studied network modifications by viral interference. The predicted system behavior is also well compatible with the experimental results obtained [11]. Our model considers the system response of six receptors: Fas, IGFR, IL-3R, IL-1R, TNF-R1 and TRAIL-R. As far as the variety of crosstalks depicted above is concerned, they show distinct responses to external signals. Furthermore, proliferation and apoptosis protein nodes are distinguishable and behave differently in states A1 to A4. A good example for this is Akt. It is active in steady states 3 and 4 and inactive in steady states 1 and 2. As a further control for the correct system implementation we considered Bid KO cells (Table 3) and the simulation result also corresponds with the experimental readout data.

**Table 3:** Steady state analysis for hepatocyte Bid KO Model (Model 5)^1^

**Model A** **Steady State A1**	**Value**	**Model A** **Steady State A2**	**Value**	**Model A** **Steady State A3**	**Value**	**Model A** **Steady State A4**	**Value**
Akt*	**0,99**	Akt*	**0**	Akt*	**0,99**	Akt*	**0**
Akt/PKB	**0,79**	Akt/PKB	**0**	Akt/PKB	**0,79**	Akt/PKB	**0**
Apaf1	**1**	Apaf1	**0**	Apaf1	**0**	Apaf1	**1**
Apoptosis	**0,98**	Apoptosis	**0,98**	Apoptosis	**0**	Apoptosis	**0,98**
Bcl-2/Xl	**0**	Bcl-2/Xl	**1**	Bcl-2/Xl	**1**	Bcl-2/Xl	**0**
Bcl-2/Xl*	**0**	Bcl-2/Xl*	**1**	Bcl-2/Xl*	**1**	Bcl-2/Xl*	**0**
Caspase-10	**0**	Caspase-10	**0,85**	Caspase-10	**0**	Caspase-10	**0,85**
Caspase-3	**0,78**	Caspase-3	**0,95**	Caspase-3	**0**	Caspase-3	**0,99**
Caspase-6	**0,98**	Caspase-6	**1**	Caspase-6	**0**	Caspase-6	**1**
Caspase-7	**0,93**	Caspase-7	**0,83**	Caspase-7	**0**	Caspase-7	**1**
Caspase-8	**0**	Caspase-8	**0,78**	Caspase-8	**0**	Caspase-8	**0,78**
Caspase-9	**1**	Caspase-9	**0**	Caspase-9	**0**	Caspase-9	**1**
Cleavage	**1**	Cleavage	**1**	Cleavage	**0**	Cleavage	**1**
CytC	**1**	CytC	**0**	CytC	**0**	CytC	**1**
DFF40	**1**	DFF40	**1**	DFF40	**0**	DFF40	**1**
DFF45	**1**	DFF45	**1**	DFF45	**0**	DFF45	**1**
Fragmentation	**0,85**	Fragmentation	**0,85**	Fragmentation	**0**	Fragmentation	**0,85**
Survival Genes	**0**	Survival Genes	**0**	Survival Genes	**0,99**	Survival Genes	**0**
Degradation	**0,93**	Degradation	**0,93**	Degradation	**0,9**	Degradation	**0,93**
Caspase	**0,01**	Caspase	**1**	Caspase	**0,01**	Caspase	**1**
IKK	**0,01**	IKK	**0**	IKK	**0,93**	IKK	**0**
IkBa	**0**	IkBa	**0**	IkBa	**0,91**	IkBa	**0**
NFkB	**0,01**	NFkB	**0**	NFkB	**0,79**	NFkB	**0**
PI3K	**0,73**	PI3K	**0**	PI3K	**0,73**	PI3K	**0**
Stat3	**0**	Stat3	**0,93**	Stat3	**0,93**	Stat3	**0**
Survival	**0**	Survival	**0**	Survival	**0,93**	Survival	**0**
mTor	**1**	mTor	**0**	mTor	**1**	mTor	**0**
s69	**0**	s69	**0**	s69	**1**	s69	**0**
tBid	**0**	tBid	**0**	tBid	**0**	tBid	**0**

^1^All interesting nodes of the network are listed together with the activation state for each steady state.

### Extensions

The strength of our model relies on the large scale exploration of networks by combining sequence/domain and topological network analyses and applying SQUAD for system state sampling. Further crosstalk behavior can be easily simulated, as well as alternative and/or larger networks. It should of course be noted, that convergence problems may occur with SQUAD regarding sampling of very large networks (over 150 nodes). The domain analysis (Additional file 2: Table 1S) allows to quickly detect phylogenetic conservation of the numerous nodes as well as key domains involved in the respective pathway and function based extension of the Boolean network. Moreover, for a detailed refined model domains and sequences can be used to check for potential modifications such as phosphorylation and the formation of protein complexes and regulatory interactions.

Enlargement of the network model did not lead to more than 4 distinctive system states. Two types of apoptotic pathways can be distinguished besides two non-apoptotic pathways (state 1). The non-mitochondrial extrinsic pathway corresponds to state 2. The intrinsic pathway is active in steady state 1. The survival pathway comes in two flavors, one involving activation by Bcl-2 and similar effectors (state 4) or without them (state 3). With regard to interfering signals, AKT clearly seems to play the role of a centrally located turntable in the system. Some AKT substrates are activated (mTOR, PI3K, MKK4 and ask1) but others are inactive (DAXX and MEKK1) in the model. Further possibilities for cross-input involve receptors or the titration of anti-apoptotic factors such as FLIP.

## Conclusion

Apoptosis of liver cells is dependent on external signals such as components of the extracellular matrix and cell-cell-contacts, which are processed by a variety of numerous nodes of which several are examined here for their system effects. Despite different input interferences and presumably also due to natural selection, the system nevertheless appears to be optimized to adopt a small number of clear and distinguishable states, and the various inputs and crosstalk mechanisms only optimize the best choice between them. In our model we found two non-apoptotic and two apoptotic states, although the degree of activation at a node can differ widely until the absolute system state is altered. The model is still an over-simplification of the complete cellular network and its different states, and operates independently of detailed kinetic data and parameters for individual nodes. Nevertheless, it allows modeling the readout of apoptosis after FasL stimulation with qualitative agreement and includes crosstalks from collagen/integrin signaling, the effect of genetic deletion of Bid and the consequences of viral infection.

The complete simulation is freely available (executable, source code, protein sequences, generated models). We are confident that our simplified hepatocyte apoptosis crosstalk model will be exploited for further investigations which should include, besides a systems biology analysis, a more detailed experimental analysis of the various crosstalks (viral infection, Bid KO, collagen/integrin crosstalk, AKT substrates, FLIP titration).

## Methods

### Network setup

For the creation of the network, we used expert knowledge and databanks such as KEGG for a collection of known network nodes to get a basis model including 66 nodes (Model C, see suppl. Material, all sequences and domains are given). To get additional nodes we used Genbank (adding nodes from literature and checking their sequence), STRING (predict edges, i.e. interactions between nodes) and network module analysis [14] to expand the network. Besides the versions shown in Table 1 a number of further modifications tested network robustness and behavior as well as system input and stability from different connections and crosstalk possibilities (see suppl. Material). The final model includes 74 nodes (Model A). Functional analysis of individual nodes includes sequence and domain analysis [15] to determine function, in particular activation and inhibition [see Additional file 2: Table 1S, Table 2S]. For the creation and visualization of the model we used Celldesigner Version 3.5.1 [16].

### Dynamic system stability analysis

The complete system for apoptosis has 74 nodes and 108 edges. This can be set up as a Boolean network, which is given here in SBML format [see Additional file 3]. We applied and used for this the output created from Celldesigner [16]. In order to systematically test the number of stable states in the network we used the program SQUAD [6] (accepts input as an xml or smbl file). With this program we converted our Boolean network into a dynamic system. In the next step SQUAD uses an efficient heuristic to calculate a reduced order binary decision diagram [7]; the required running time for convergence in each simulation was only 2 minutes on a 2.16 GHz Intel Core 2 Duo PC. Furthermore, the program converts the network into a continuous dynamical system based on ordinary differential equations. SQUAD can operate here also in the absence of detailed kinetic parameters as it simply interpolates a hysteresis curve (exponential function) between the states completely "on" or completely "off" for each node. As shown previously [6], this allows us to identify the number and features of the stable systems states even in rather complex networks without detailed knowledge of the kinetic parameters. SQUAD therefore calculates first the steady states found in the discrete Boolean network model and then uses these states as a guide to localize the stable steady states in the continuous model. The complete set of equations is automatically generated by SQUAD upon reading in the SMBL file given in Additional file 2: Table 3S and also available from the authors on request. For validation and comparisons, values for individual nodes corresponding to different system states as well as transition values and curves were directly read out and calculated from SQUAD and plotted (see results).

## Authors' contributions

NP: SQUAD implementation and simulations, analysis of data, testing and changing different models, network analysis, writing of the manuscript. DW, KF: Experiments on apoptosis, analysis of experimental data. RS, ME, OS, JT: Modeling expertise, new input and suggestions also from own modeling projects. CB: Lead and guided the experimental part of the study, apoptosis expertise, writing of the manuscript. TD: Lead and guided the study, analysis of data and simulations, testing and changing different models, network analysis, writing of the manuscript. All authors read and approved the final version of the manuscript

## Supplementary Material

Additional file 1 **Sequence and domain analysis of the hepatocyte apoptosis network**. All protein sequences of the proteins used in the model (alphabetically sorted). Click here for file

Additional file 2 **Additional Tables and Figures**. Supplementary Tables and Figures. Click here for file

Additional file 3 **XML-formatted model**. XML version and format of the model. Click here for file
