# Supplementary material for: Modeling system states in liver cells: Survival, apoptosis and their modifications in response to viral infection
Source: BMC Syst Biol. 2009 Sep 22;3:97. doi: 10.1186/1752-0509-3-97 (PMC2760522; doi:10.1186/1752-0509-3-97)
Supplement: Additional file 2 — Additional Tables and Figures. Supplementary Tables and Figures. [file 1752-0509-3-97-S2.doc]

**Additional File 1**: file format: DOC

Title: **Additional Tables and Figures**

Description: Supplementary Tables and Figures

*Sequence- and domain analysis of individual nodes:*

**Table 1S: Activating and inhibiting nodes** (and involved domains).

**Table 2S: Individual sequences for each node included in the model** (for human hepatocytes) (additional file 2).

**Figure 1S: Interaction map highlighting the active nodes for steady state 1**

**Figure 2S: Interaction map highlighting the active nodes for steady state 2**

**Figure 3S: Interaction map highlighting the active nodes for steady state 3**

**Figure 4S: Interaction map highlighting the active nodes for steady state 4**

*Boolean network:*

**Table 3S:** **Boolean network for apoptosis and crosstalk in hepatocytes (SBML format)** (additional file 3).

**Table 4S: Steady state analysis for viral infection.**

**Table 5S: Evidence of robustness**

*Experimental procedures:*

**DEVDase caspase-3/-7 activity assay**

**SDS-PAGE and Western Blot analysis**

*Simulations:*

**Equation used by SQUAD to perform the simulation**

*Sequence- and domain analysis of individual nodes:*

Table 1S: **Activating and inhibiting nodes^1^**

^1^Color code: Mammalian domains are colored in yellow, key domains for apoptosis in red (DED, Death, CARD), kinase signaling in green, executioner caspases in black (bold). Detailed data on phylogenetic comparison are available on request.

Complex formation: Follow the network graph shown in Fig. 1. All sequences are given below in Table 2S. Note that also for these detailed modifications data are available on request.

Figure 1S**: Interaction map highlighting the active nodes for steady state 1. Active nodes a colored in yellow.**

**
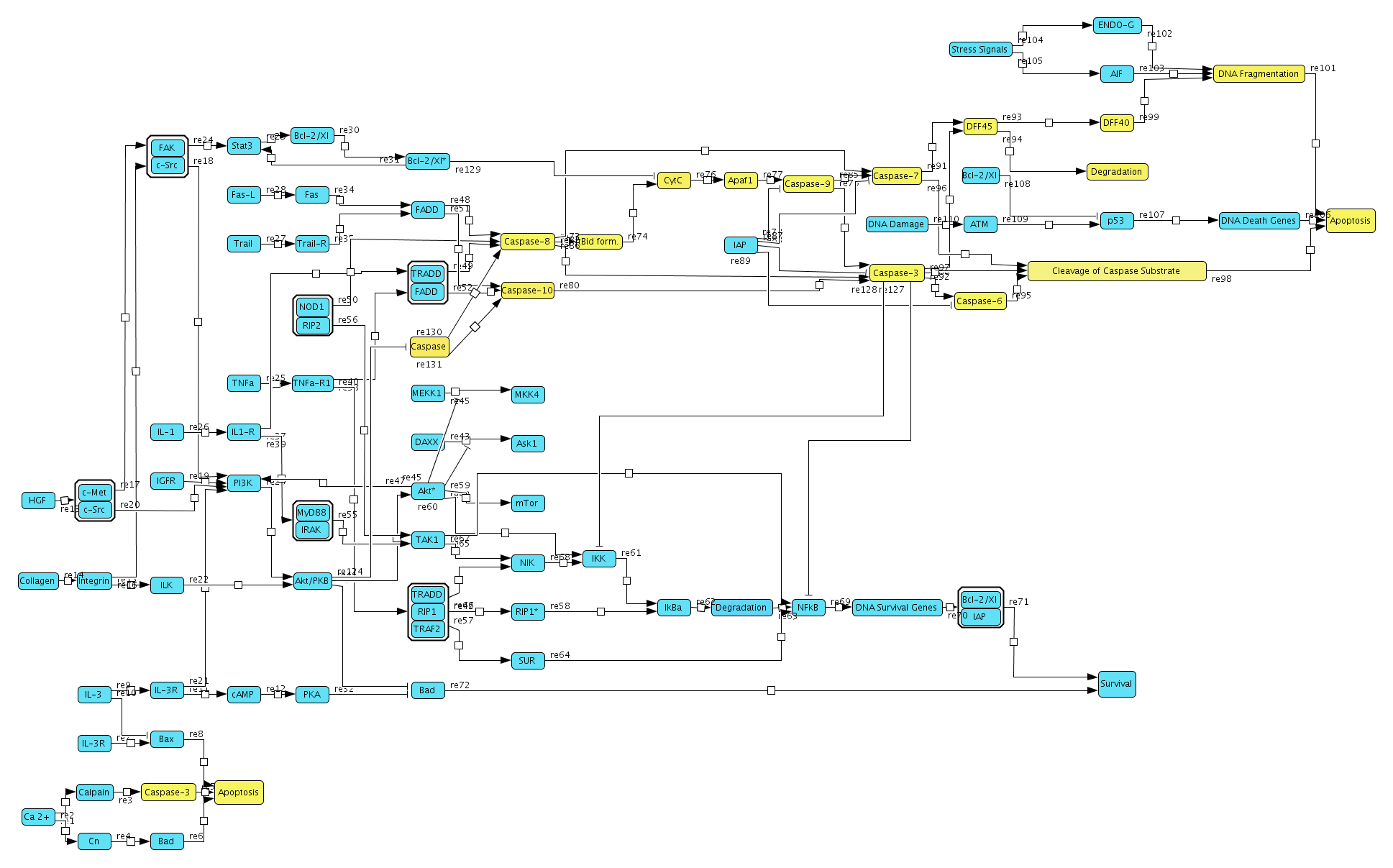
**

Figure 2S: **Interaction map highlighting the active nodes for steady state 2. Active nodes are colored in red.**

**
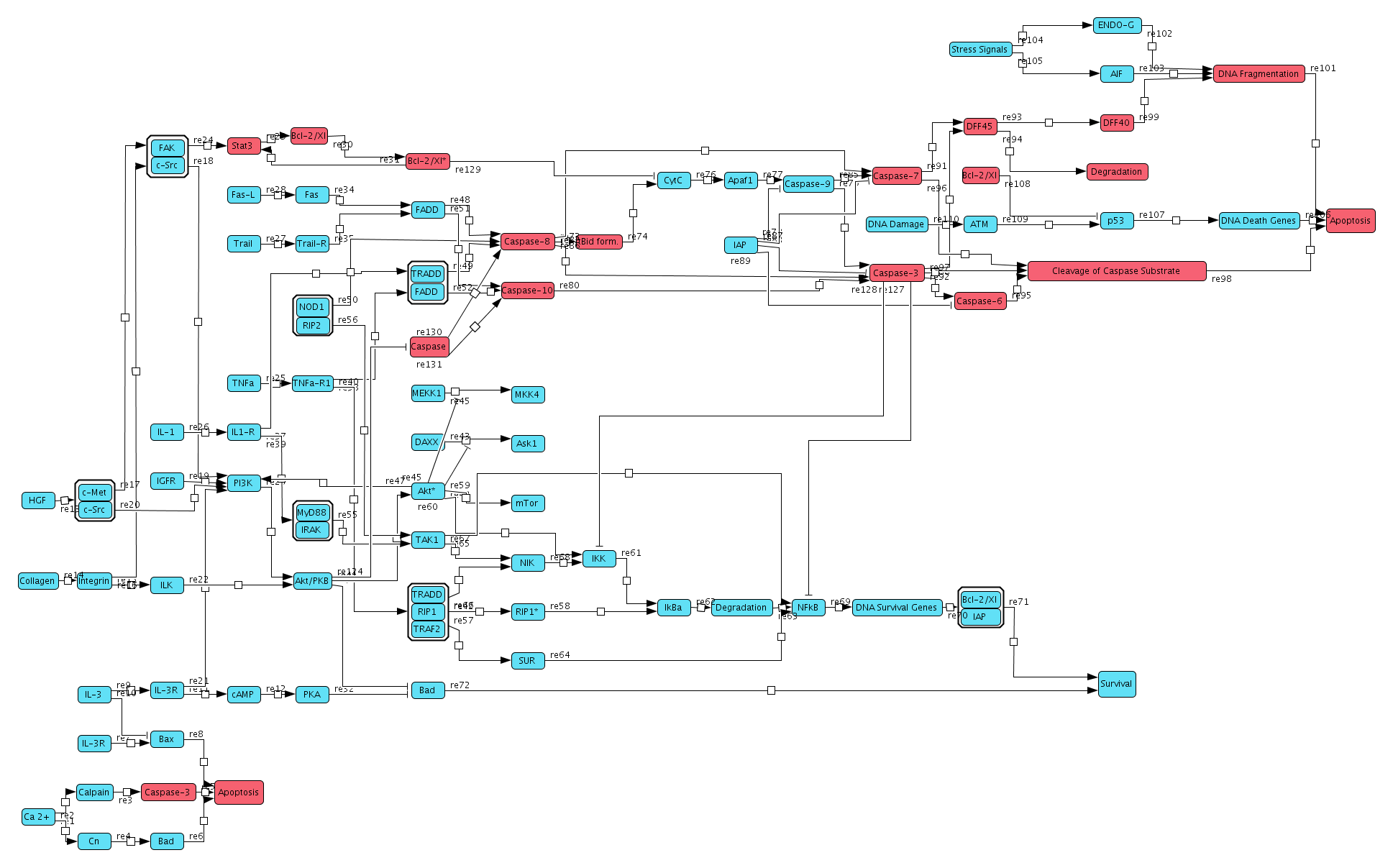
**

Figure 3S: **Interaction map highlighting the active nodes for steady state 3. Active nodes are colored in green.**

**
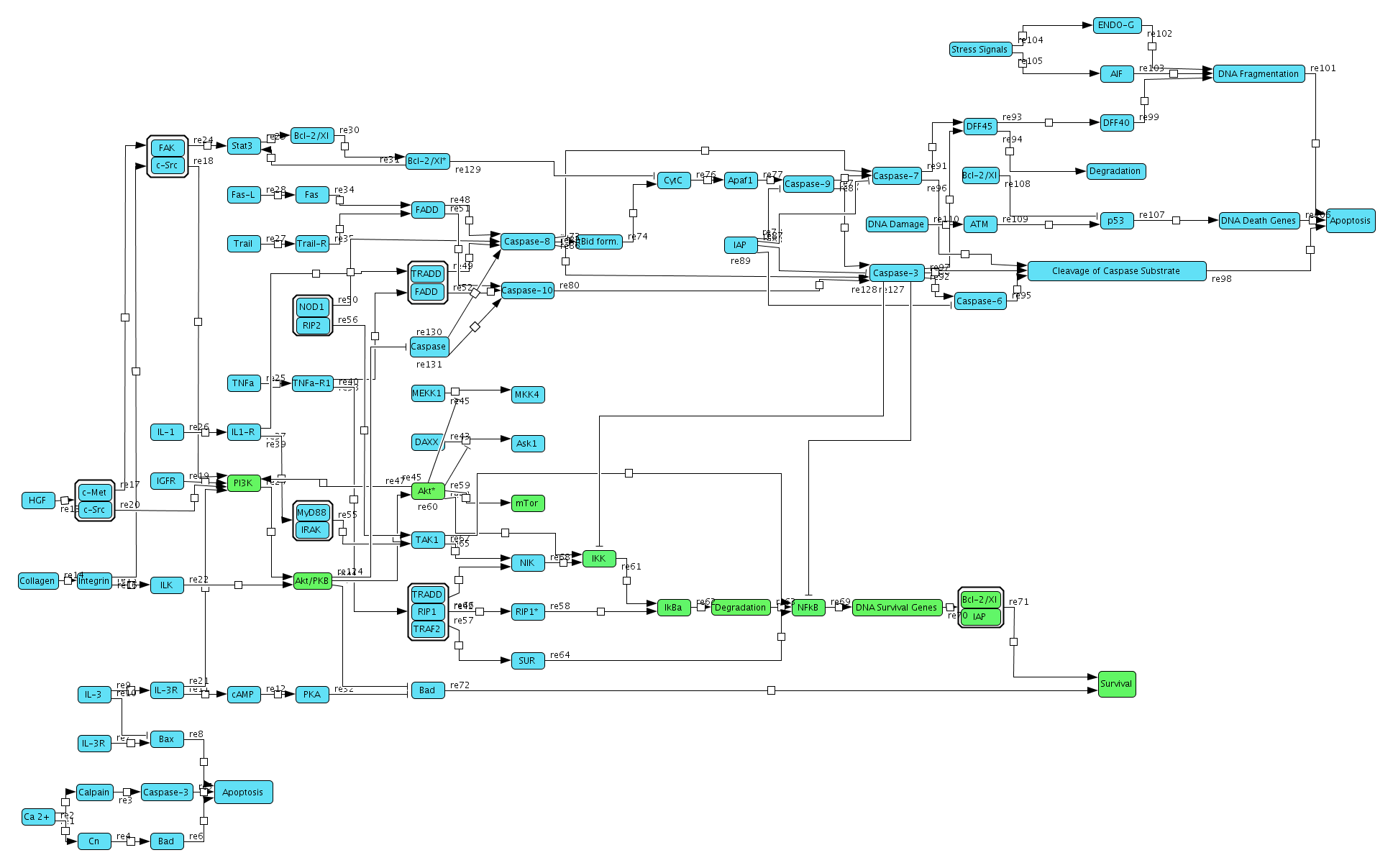
**

Figure 4S: **Interaction map highlighting the active nodes for steady state 4. Active nodes are colored in orange.**

**
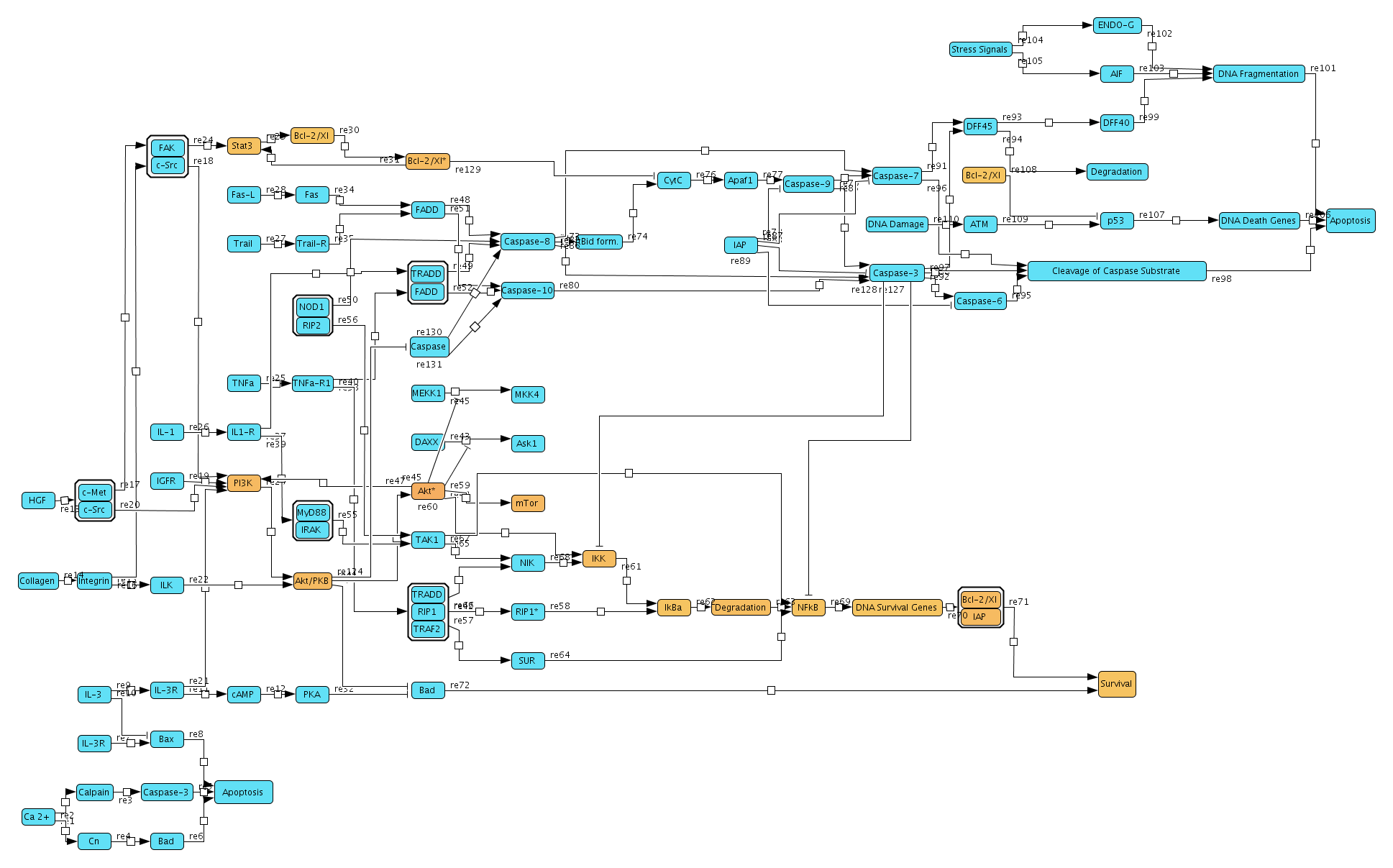
**

*Boolean network:*

Table 4S: **Steady state analysis for viral infection^2^**

^2^ Model M36 (Model 7). All interesting nodes of the network are listed together with the activation state for each steady state.

Table 5S: **Evidence of robustness**

**Reduced core model C:** This model contains only components listed in official databases, has no feedback loops and shows only one steady state. This state is an apoptotic state.

**Model 2:** We included the ILK pathway, which is important for Integrin signaling as well as the activation of Akt to the reduced core model by introducing an additional positive feedback loop and observed two different steady states: one survival state and one apoptotic state.

**Model 3:** In this model we added only TRADD/FADD interactions as well as the ILK pathway, but without introducing a new feedback loop. Only the one steady state from core model C was observed.

**Model 4:** Here the activation of Akt and the PI3K pathway were added, forming a new positive feedback loop. According to model 2 we observed the same two steady states.

**Model A:** In model A all interactions and crosstalk possibilities were considered. These include additional components as a positive feedback loop between Akt/PKB and PI3K, additional components as a positive feedback loop between Bcl-2/x_L_ and Stat3 as well as additional Akt substrates. Model A contains two positive feedback loops and shows 4 steady states: two survival states and two apoptotic ones. For growth on collagen, cytochrome c is not released (no activation by tBid, inhibition by Bcl-2/x_L_) and apoptosis is delayed (both apoptosis nodes in the model, actually a molecular cascade).

*Experimental procedures:*

**Isolation and cultivation of primary mouse hepatocytes**

Primary mouse hepatocytes were isolated from 6-12 week old C57BL/6 mice and cultured on collagen-I dishes as described in [6]. For apoptosis, the cells were treated with 50 ng/ml of FasL and various apoptotic parameters were measured as described in [6]. Hepatocytes in suspension were used for apoptosis assays directly after isolation without plating on dishes. For this purpose, the cells were treated with FasL or left untreated and carefully shaken in a 5% CO_2_ incubator at 37 °C for different time points.

**Antibodies and reagents**

Rabbit anti-caspase-3 (9661 and 9662) and anti-caspase-9 antibodies (9504) were obtained from Cell Signaling and mouse anti-caspase-8 antibodies (1G12) from Alexis. Mouse anti-cytochrome c antibodies (7H8.2C12) were purchased from BD Biosciences. Horseradish peroxidase-conjugated goat anti-rabbit, goat anti-rat and goat anti-mouse IgG antibodies were purchased from Jackson Immuno Research Laboratories. The fluorogenic caspase-3 substrate Ac-DEVD-AMC was purchased from Alexis, high-grade collagen I and the DNA-specific dye propidium-iodide (PI) from Sigma and Hoechst 33342 from Molecular Probes. Recombinant Fc-FasL was kindly provided by Dr Pascal Schneider, Institute of Biochemistry, Lausanne.

**Preparation of cytosolic and mitochondrial lysates**

For the preparation of cell lysates, 1 x 10^6^ primary hepatocytes were treated with a defined amount of recombinant Fc-FasL. Suspension hepatocytes were treated immediately after isolation. At selected time points, the cells were detached with 0.375% trypsin, supplemented with 1 µM EDTA, for 2-3 min (without resuspending) followed by two washing steps in WME and PBS. Cytosolic fractions were prepared by resuspending the cell pellets in homogenization buffer (25 mM HEPES-KOH, pH 7.4, 2 mM EGTA, 2 mM MgCl_2_), supplemented with protease inhibitors (10 µg/ml aprotinin, 50 µg/ml leupeptin, 400 ng/ml pepstatin, 5 µg/ml cytochalasin B, 100 µM PMSF and 10 mM DTT, followed by several freeze-thawing cycles and ultracentrifugation at 112’000 x g for 30 min. The supernatant was used for DEVDase caspase-3 activity assays and Western blotting. For the preparation of crude mitochondrial fractions, detached hepatocytes or liver pieces were resuspended in SEM buffer (10 mM Hepes-KOH and 250 mM sucrose) containing protease inhibitors as described above and homogenized in a Dounce homogenizer. The nuclei were removed by centrifugation at 500 x g and crude mitochondria were obtained from the postnuclear supernatant by an additional centrifugation step at 10’000 x g (mitochondria or P_10_). Mitochondria were washed twice in SEM buffer and then solubilized in H8 buffer (20 mM Tris-Hcl, pH 7.5, 2 mM EDTA, 2 mM EGTA, 6 mM ß-mercaptoethanol) containing 1% SDS. The post-mitochondrial supernatant (cytosol or S_10_) was concentrated by Centricon Ultrafiltration and used, together with the mitochondrial fraction for Western blot analysis.

**DEVDase caspase-3/-7 activity assay**

Caspase-3 and -7 (DEVDase) activity was measured in cytosolic extracts of primary hepatocytes or MEFs, by using a fluorogenic assay, as described [6]. Alternatively, caspase-3/-7 was measured by the luminescence caspase-3/-7 Glo^TM^ assay, as described by the manufacturer (Promega).

**SDS-PAGE and Western Blot analysis**

Equal amounts of protein (60 – 80 µg), isolated by sub-cellular fractionation as described above, were electrophoresed on 12% or 15% SDS-PAGE gels, transferred to PVDF membranes, probed with different antibodies and visualized by enhanced chemiluminescence (PIERCE). Anti-caspase-3 antibodies were used at 1:500, anti-caspase-8 antibodies at 1:1000, anti-caspase-9 antibodies at 1:500, anti-Bid antibodies at 1:700 and anti-cytochrome c antibodies at 1:500.

**MTT viability assay**

After exposing to FasL for different time periods, primary hepatocytes were treated on the plate with 0.5 mg/ml MTT (Sigma) dissolved in PBS, and incubated at 37 ºC until a color change (purple) was observed. The plate was shortly centrifuged, the supernatant removed and the cells dissolved in DMSO. The samples were transferred into a fresh 96-well plate, and the color reaction measured with an ELISA reader at 595 nm.

*Simulations:*

**Equation used by SQUAD to perform the simulation:**

SQUAD automatically converts the static network into a continuous dynamical system using the following equation.


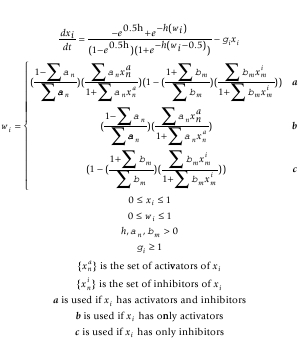


By default, SQUAD sets all values of α's (weight of activations), β's (weight of inhibitions) to 1, and a value of 10 to h (the gain of the sigmoid).
